# Current nest box designs may not be optimal for the larger forest dormice: Pre‐hibernation increase in body mass might lead to sampling bias in ecological data

**DOI:** 10.1002/ece3.8437

**Published:** 2021-12-06

**Authors:** Hesamaddin Farhadi

**Affiliations:** ^1^ Faculty of Sciences University of Guilan Rasht Iran

**Keywords:** forest dormouse, gnawing behavior, nest box design, occupancy, sampling bias, seasonal variation in body weight

## Abstract

1. Biologists commonly use nest boxes to study small arboreal mammals, including the forest dormouse (*Dryomys nitedula*). Hibernating dormouse species often experience pronounced seasonal variations in body mass, which might lead to sampling biases if it is not taken into account when designing nest boxes. In my study of the forest dormouse, I noticed that the entrance hole of nest boxes had been gnawed. I hypothesized that this behavior was exhibited by the individual dormice of higher body mass, who were unable to pass through the entrance holes.
2. To test my hypothesis, I categorized the individual dormice present inside nest boxes based on their body mass and then compared the seasonal body mass dynamics with the timing of the gnawing behavior. I also compared nest box occupancy by the forest dormouse before and after the gnawing behavior.
3. Interestingly, I found that the gnawing behavior was displayed exclusively when part of the dormouse population increased considerably in body mass, which supports my hypothesis. Additionally, nest box occupancy decreased significantly from 20% before to 4.6% after the gnawing behavior.
4. I suggest that researchers include nest boxes with entrance holes larger than 4 cm in future studies of the forest dormouse to prevent the possible exclusion of the conspecifics that have higher body mass before hibernation. This type of sampling bias might also concern studies of other species, such as the fat dormouse, that similarly show pronounced seasonal variations in body mass. I recommend that biologists consider the seasonal body mass dynamics of the target species when designing nest boxes to minimize bias in ecological data and improve management actions.

## Graphical abstract

In this paper, we attempt to reveal why forest dormice gnawed on the entrance holes of nest boxes. Our data on seasonal variation in body mass show that this behavior was exhibited by individual dormice of higher body mass before hibernation, who were unable to pass through the entrance holes. This indicates not only are currently commonly used nest box designs not optimal for the larger forest dormice, but also this unsuitable design might reduce the sampling probability of these conspecifics, which will lead to a sampling bias. This effect is not probably limited to the species and is likely to happen in nest box studies of other cavity‐nesting mammals. In addition to improving ecological research and inference, our findings can inform management programs that use nest boxes to increase nest site availability for cavity‐nesting mammals.

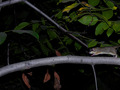

## 1 INTRODUCTION

Biologists have long used artificial nest boxes as a convenient tool for obtaining occupancy, abundance, and reproductive data for various taxa, including birds and small mammals (e.g., Goldingay, Quin, et al., 2020; Menkhorst, 1984; Monti et al., 2019; Williams et al., 2013). Obviously, nest boxes should be specifically designed to suit the goals of an intended study. It is important to ensure that nest box design is tailored to species‐specific requirements (Zingg et al., 2010; see also Goldingay, Rohweder, et al., 2020). For example, a nest box designed for a small sugar glider would not be efficient for studying a much larger bobuck (Menkhorst, 1984).

While the average characteristics, such as size, of a species are presumably the first factors involved in determining the optimal nest box dimensions, there can be variations within a species, which can further complicate the case. More specifically, individuals of a species might seasonally change in body mass because of changes in physiological demands and food intake. Although this type of seasonal variation is minor in many species, some show a highly dynamic body mass. In such cases, if the dimensions of a nest box, particularly the entrance hole diameter, are designed based on the average body mass of a species at its minimum, it may not be suitable for when body mass is at a maximum. In the worst scenario, this may lead to sampling biases in ecological data due to the uselessness of nest boxes in certain periods, for certain individuals of the population, or both.

No previous studies have investigated the possible biases in ecological data that result from nest boxes designed improperly for a species with high seasonal variations in body mass. However, researchers have recently described some of the ways in which nest box design can considerably affect the ecological processes and phenomena under study. Clark et al. (2020) observed that the American kestrels experience a significantly higher prey delivery failure rate at the boxes with small holes compared to boxes with large holes. Møller et al. (2014) reported that there was a significant, positive relationship between clutch size and the base area of the nest box. Moreover, Saunders et al. (2020) found Carnaby's black cockatoos (*Calyptorhynchus latirostris*) produced chicks of smaller mass in artificial hollows of smaller volume. Nevertheless, for example, a considerable proportion of ecological studies on birds of prey using nest boxes in the Northern Hemisphere before 2012 have not provided any details on nest box design such as size, shape, and material (Lambrechts et al., 2012), which makes it impossible to compare such studies and draw conclusions on the effects of nest box design on ecological studies.

Since the forest dormouse (*Dryomys nitedula*), an arboreal rodent, has a highly seasonally variable body mass, nest boxes used to study this species must be designed with more caution. Most of the debates thus far over the potential effects of nest box design on the outcomes of biological studies have focused on birds, which often show relatively small temporal variation in body mass of adults (e.g., Haftorn, 1989; Lehikoinen, 1987; Wu et al., 2014). By contrast, some species of Gliridae show a quite pronounced change in body mass during activity season. For example, both the forest dormouse and the fat dormouse (*Glis glis*) can nearly double in body mass from the beginning of activity season to just before hibernation (Fietz et al., 2005; Juškaitis, 2015). This highlights the importance of designing nest boxes that are suitable for the target species throughout the year.

In my study of the forest dormouse using nest boxes, I noticed that the entrance holes of some of the deployed nest boxes had been gnawed. This behavior has been reported for some mammals (Goldingay et al., 2015; Le Roux et al., 2016), but not for the forest dormouse. I hypothesized that this behavior was exhibited by the individual dormice of higher body mass, who were unable to pass through the entrance holes. In other words, I argue that nest boxes used in ecological studies of the forest dormouse must have a larger entrance hole to prevent the possible exclusion of conspecifics based on their body mass, thus yielding less biased data. If the hypothesis is correct, the frequency of the gnawing behavior should be greatest in the season when dormice have higher body mass. Ultimately, this increase in weight might also lead to a reduction in nest box occupancy by the forest dormouse in the same period. To test my hypothesis, I compared the seasonal body mass dynamics of the forest dormouse with the timing of the gnawing behavior. I also compared nest box occupancy by the forest dormouse before and after the gnawing behavior.

## 2 MATERIALS AND METHODS

**FIGURE 1:**
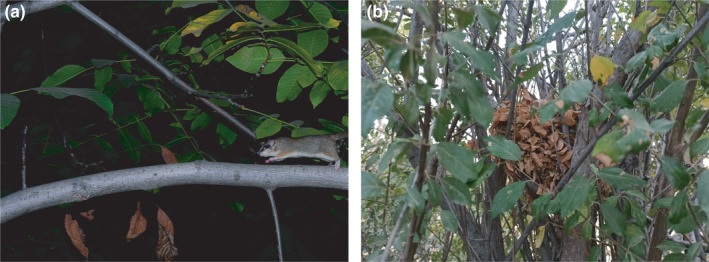
(a) A forest dormouse (*Dryomys nitedula*) in Zanjan province, Iran. (b) The nest of a forest dormouse between the branches of a tree in my study area

This field study was conducted in three plots within a study area located in the east of Zanjan province, Iran, and to the south of the Alborz Mountain Range (elevation 1,600 m). The study area consisted of private lands with agricultural land use. Other native species than the forest dormouse that could theoretically occupy nest boxes include the Caucasian squirrel (*Sciurus anomalus*), the beech marten (*Martes foina*), songbirds, the great spotted woodpecker (*Dendrocopos major*), and other bird species, although the dimensions of my boxes seem to be too small for the Caucasian squirrel. Squirrels and woodpeckers might in particular gnaw on the box entrances. The forest dormouse (Figure 1a) is the only dormouse species inhabiting the study area.

**FIGURE 2:**
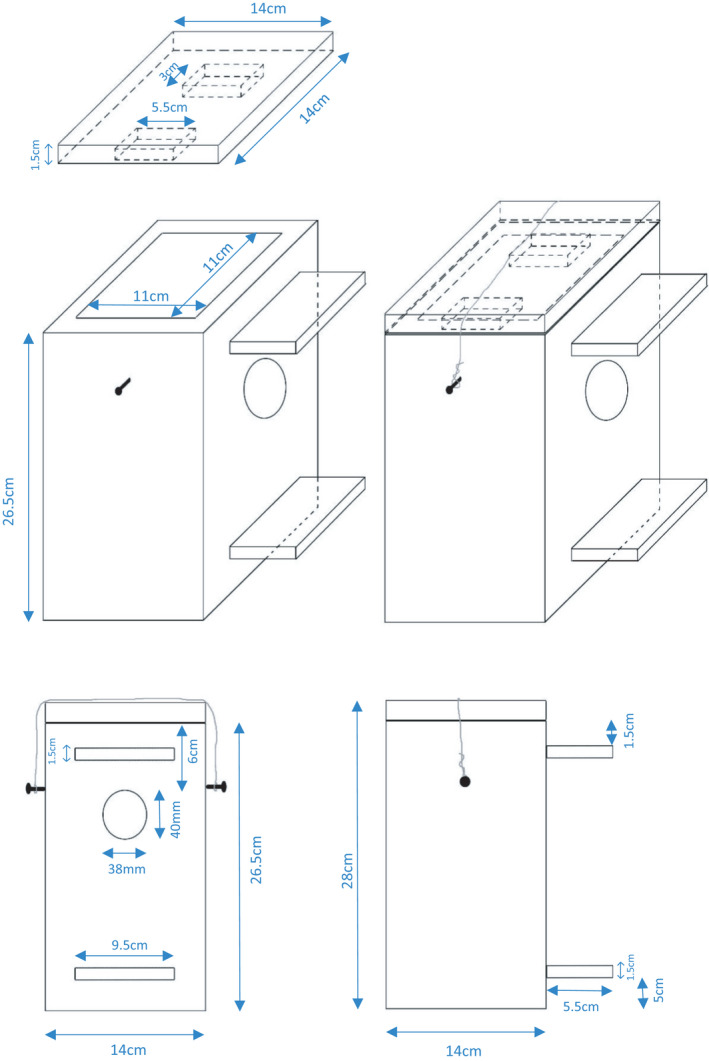
The dimensions and design of the nest boxes used in the present study

In mid‐April 2019, I deployed 65 nest boxes across the study area, of which 42 were in plot one, 11 in plot two, and 12 in plot three. The approximate areas of plots 1, 2, and 3 were 60,000, 8,000, and 10,000 m^2^, respectively. The plots were ca. 500–1,100 m apart. I wired the nest boxes to trees >20 m apart, at a height of 1.5–3.5 m. The internal dimensions of nest boxes were 25 * 11 * 11 cm, the same as suggested by Juškaitis (2015). However, the entrance hole was oval, with 38 and 40 mm diameters (Figure 2), contrary to the 35 mm reported by Juškaitis. The nest boxes were constructed from black poplar wood.

**FIGURE 3:**
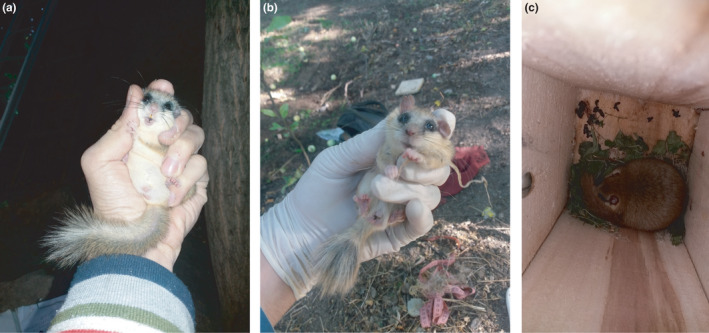
(a) An overweight and (b) an underweight forest dormouse captured inside nest boxes (both are male); note the substantial fat accumulation in the pelvic and abdominal regions of the overweight dormouse. (c) Another overweight dormouse inside a nest box (the internal basal dimensions of the nest box are 11 * 11 cm)

I visited the nest boxes six times during daylight every 4–6 weeks from May to November 2019 and recorded the presence of the forest dormouse inside nest boxes. The species is known to give birth between June and July (Juškaitis, 2015; Paolucci et al., 1989), although there might be geographic variations. During the visits, I captured the dormice present inside nest boxes and visually categorized them as underweight, overweight, and uncategorized (Figure 3). Uncategorized individuals were those who escaped before I could capture them or confidently assign them a weight category. I used these categories only as a relative measure of body mass, not to convey health condition. I considered an individual dormouse overweight if it had a substantial fat reserve. I preferred visual body mass categorization over the use of a weighing scale to minimize disturbance and handling time. This was also because capturing dormice was not always successful, especially when there were several individuals in a single nest box. In such situations, I could at least visually determine the body mass category of some of the uncaptured dormice before they escaped, resulting in a larger sample size. Fieldwork procedures were in accordance with the mammalogical procedure established by the American Society of Mammalogists Animal Care and Use Committee (1998). I pooled data from the three plots to achieve a larger sample size.

I conducted a McNemar test to see whether nest box occupancy changed from before to after the gnawing behavior (i.e., from the fifth to the sixth visit). I chose this test over chi‐square because my data groups were paired (Adedokun & Burgess, 2011; Hoffman, 1976). My categorical independent variable was the visit occasion, with two groups corresponding to the fifth and sixth visits. My categorical dependent variable was dichotomous, with two categories of occupied and unoccupied. I considered a nest box occupied only if one or more dormice were present in the nest box at the time of visit. I used R version 4.1.1 for analysis (R Core Team, 2021).

## 3 RESULTS

**FIGURE 4:**
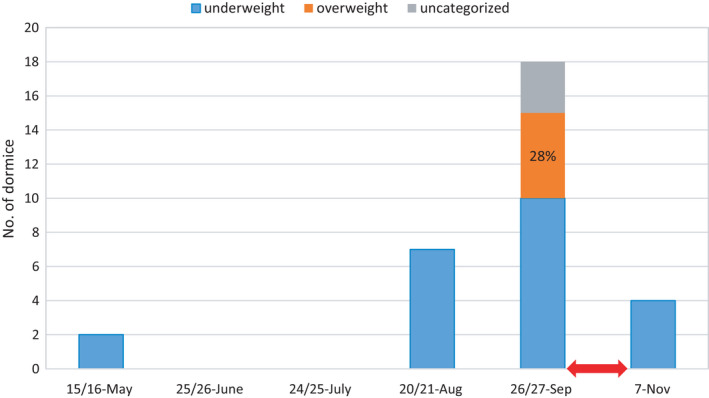
Proportion of different body mass categories of the forest dormouse present inside nest boxes in different months of year. The red arrow marks the period when the entrance holes were gnawed. I inspected 65 nest boxes from May to November 2019

The number of dormice observed inside nest boxes ranged from zero in the second and third visits to a maximum of 18 in the fifth visit (Figure 4). Although no dormice were immediately present in nest boxes in the second and third visits, I still found indirect evidence of dormouse presence (new nests, feces, or food remains) in the same visits. Except in the fifth visit, all of the individual dormice captured inside nest boxes were underweight. However, out of the 18 dormice captured in the fifth visit, 10 were underweight (~55%), 5 overweight (~28%), and 3 uncategorized (~17%) due to unsuccessful capture of the animals. The number of dormice observed fell from 18 in the fifth visit to 4 in the sixth visit.

**FIGURE 5:**
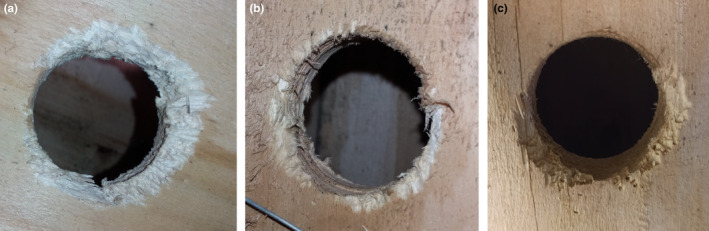
Gnaw marks of the forest dormouse around the entrance holes of nest boxes. The species has gnaw marks that are not similar to any other sympatric species'

Only in the sixth visit, did I notice that the entrance holes of six nest boxes were gnawed (Figure 5), indicating it occurred exclusively in the intervening period. My data support the prediction that the frequency of the gnawing behavior is maximized in the season when at least part of the forest dormouse population increases considerably in body mass. It is noteworthy that despite being gnawed, the diameter of the entrance holes had changed only a few millimeters (<5 mm) and that the six gnawed nest boxes were located in plot one.

Additionally, nest box occupancy decreased significantly from 20% in the fifth visit to 4.6% in the sixth visit, *χ^2^* (1, *N* = 65) = 6.75, *p* = .009.

## 4 DISCUSSION

In general, the present study confirms the hypothesis that the forest dormice tend to gnaw on the entrance holes of nest boxes when they have a higher body mass before hibernation and thus face difficulty passing through the entrance holes. It is a common finding that animals will gnaw on nest box entrances in an attempt to gain access (see Goldingay et al., 2007). My time‐series data on the proportion of different body mass categories are consistent with the findings of Juškaitis' study (2015), in which body mass is measured using scales and is maximized before hibernation season.

**FIGURE 6:**
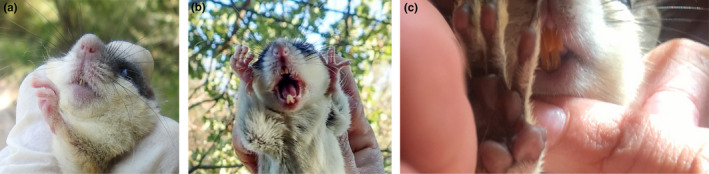
Upper and lower incisor teeth of the forest dormouse (a & b) and the Caucasian squirrel (c). The former has needle‐like lower incisors while the latter has lower incisors that are as wide as the upper ones (photographs b by Jon Hall from Mammal Watching and c by Mahmoud Keshvari)

Considering the local fauna, it is quite evident that the gnawing behavior was displayed by the forest dormouse, not other species. In the first place, the shape and the size of upper and lower incisors deduced from the chew marks best matched that of the forest dormouse. The forest dormouse, compared to the sympatric Caucasian squirrel, have thinner and more needle‐like lower incisors (Figures 5 and 6) and their upper incisors leave a V‐shaped chew mark (based on my field inspections of the captured dormice). Other native taxa that may be, at least partly, arboreal include mustelids, felids, and birds, none of which are suspected of gnawing on the boxes in view of the chew marks.

The only logical explanation for this behavior seems to be that dormice gnawed on the entrance holes of nest boxes to make them larger, and thus more suitable, while they were experiencing an increase in body mass before hibernation. I am quite certain that this behavior is not a sign of wood consumption as a food source. Firstly, previous diet analyses of the forest dormouse did not report wood as a food item for the species (Juškaitis & Baltrūnaitė, 2013; Nowakowski & Godlewska, 2006). Dormice (Gliridae) lack a caecum and are less adapted to digest cellulose using enteric symbionts than other small mammals (Vorontsov, 1967, as cited in Juškaitis & Baltrūnaitė, 2013), which further disproves wood ingestion by the species. Secondly, as indicated earlier, gnawing occurred only at a specific time of year. Thirdly, dormice gnawed on no other parts of the nest boxes than the entrance holes.

I am uncertain as to why dormouse occupancy of nest boxes dropped significantly during the same period that the gnawing behavior was displayed. Juškaitis (2015) also reported that, in Lithuania, the majority of adult dormice left the nest boxes in late August. I propose three explanations: First of all, the forest dormouse might naturally prefer to hibernate in the subcanopy, the floor, or the ground, rather than in the canopy, where I placed most of my nest boxes (see Figure 1b and Juškaitis et al., 2012; Skahan, 2004; Shibata et al., 2004; Figarski, 2009; Ściński & Borowski, 2006; Andreychev, 2021 for descriptions of shelter sites of dormice other than nest boxes). These microhabitats may provide better thermal insulation or protection against harsh winter conditions, predation, or anthropogenic disturbances. Secondly, nest boxes that are disturbed by repeated visits might not be adequately safe for hibernation. The third explanation is that dormice might start to leave nest boxes on account of unsuitable nest box design, including small entrance hole, for when they are experiencing a major pre‐hibernation increase in body mass. I cannot confidently prove or reject any of the three explanations using the data obtained in my study. However, the absence of overweight individuals in the sixth visit suggests that the third explanation might be, at least partly, true, although the low count of dormice in the sixth visit makes it impossible to draw a strong inference. Interestingly, adult dormice, in Lithuania, left nest boxes earlier than juveniles (Juškaitis, 2015), which might be because juveniles increase in body mass later than adults.

If narrow entrance holes are responsible for even a small proportion of the decline in occupancy, this implies that the currently commonly used nest box designs inevitably introduce a sampling bias into the ecological studies of the forest dormouse. Put simply, conspecifics with higher body mass might have a lower sampling probability. Even though this is only a possibility, I suggest that it is wise to include nest boxes with entrance holes larger than 4 cm in diameter to prevent the possible exclusion of the individual forest dormice with seasonally high body mass. However, if entrances are too large, then larger species might exclude the dormice. “How much larger is best?” is a question that needs to be answered in further comparative experiments where dormice are offered a range of nest box entrance sizes.

Another point that should be considered about this study is that there is notable geographic variation in body mass of the forest dormouse (see Juškaitis, 2015; Stubbe et al., 2012). Dormice from northern habitats tend to be of lower body mass, probably due to shorter activity season, and as a result, they might not need larger entrance holes.

In conclusion, unsuitable nest box design may, in some cases, be a hidden source of bias, yet it is overlooked in many studies. One potential source of such bias can be intraspecific, seasonal biological differences. I recommend that researchers and managers consider seasonal variations in the body mass of the target species when designing nest boxes to minimize bias in ecological data and improve management actions. This can be achieved simply by offering a range of nest box designs to the target species.

## CONFLICT OF INTEREST

None.

## AUTHOR CONTRIBUTION

**Hesamaddin Farhadi:** Conceptualization (lead); data curation (lead); formal analysis (lead); investigation (lead); methodology (lead); project administration (lead); resources (lead); software (lead); supervision (lead); validation (lead); visualization (lead); writing – original draft (lead); writing – review and editing (lead).

### OPEN RESEARCH BADGES

This article has earned an Open Data, for making publicly available the digitally‐shareable data necessary to reproduce the reported results. The data is available at https://doi.org/10.5061/dryad.sj3tx9664 and https://datadryad.org/stash/share/_adguv3‐V6ndzf5YWfFqSez‐00OTAwIWHpXtYUe‐54k.

## Data Availability

All data not presented in the manuscript are available from the Figshare Digital Repository: https://doi.org/10.6084/m9.figshare.17072036.v1 (Farhadi, 2021).
